# Targeting IAP proteins in combination with radiotherapy

**DOI:** 10.1186/s13014-015-0399-3

**Published:** 2015-04-26

**Authors:** Simone Fulda

**Affiliations:** Institute for Experimental Cancer Research in Pediatrics, Goethe-University, Komturstr. 3a, 60528 Frankfurt, Germany; German Cancer Consortium (DKTK), Heidelberg, Germany; German Cancer Research Center (DKFZ), Heidelberg, Germany

## Abstract

The efficacy of radiotherapy critically depends on the activation of intrinsic cell death programs in cancer cells. This implies that evasion of cell death, a hallmark of human cancers, can contribute to radioresistance. Therefore, novel strategies to reactivate cell death programs in cancer cells are required in order to overcome resistance to radiotherapy. Since Inhibitor of Apoptosis (IAP) proteins are expressed at high levels in multiple cancers and block cell death induction at a central point, therapeutic targeting of IAP proteins represents a promising approach to potentiate the efficacy of radiotherapy. The current review discusses the concept of targeting IAP proteins in combination with radiotherapy.

## Introduction

The antitumor activity of radiotherapy largely depends on the activation of a cell-intrinsic program of cell death. There are several forms of programmed cell death and apoptosis represents one of the best characterized modes of programmed cell death that plays an important regulatory role during various physiological and pathophysiological processes [1]. Cancer cells are characterized by the inability to undergo cell death [2]. This evasion of apoptosis contributes not only to carcinogenesis and progression of tumors, but also to resistance to various current therapies including radiotherapy [3]. This implies that it will be critical to find new ways to overcome apoptosis resistance in order to improve the efficacy of radiotherapy. One strategy resides in antagonizing antiapoptotic mechanisms, thereby lowering the threshold for the induction of radiotherapy-mediated cell death. The current review focuses on targeting IAP proteins, a family of antiapoptotic proteins that play a critical role in the regulation of sensitivity and resistance of cancer cells.

### IAP proteins as therapeutic targets for radiosensitization

The family of IAP proteins comprises eight human members among which X-linked Inhibitor of Apoptosis protein (XIAP) possesses the most pronounced antiapoptotic activity via binding to and inhibiting caspase-3, -7 and -9 [4]. Caspases are a family of proteases that play a critical role as effector molecules of apoptosis [5]. Upon activation, for example via proteolytic cleavage of their pro-enzyme forms, caspases cleave a large variety of substrates and effector caspases including caspase-3 and -7 which are known as central effector molecules of apoptotic cell death. In addition to blocking caspase activation, IAP proteins can disable the induction of cell death via their Really Interesting New Gene (RING) domain with E3 ligase activity, which is responsible for the ubiquitination and subsequent degradation of apoptosis-regulatory factors by the proteasome [4]. Also, the E3 ligase activity of IAP proteins, e.g. of XIAP and cellular Inhibitor of Apoptosis (cIAP) proteins, is involved in the modulation of Nuclear Factor-kappaB (NF-κB) activation [4]. While cIAP1 and cIAP2 promote activation of the canonical NF-κB pathway by non-degradative ubiquitination of the serine/threonine kinase receptor-interacting protein (RIP)1, they limit non-canonical NF-κB signaling by mediating the constitutive proteasomal degradation of NF-κB-inducing kinase (NIK), a kinase that initiates signaling in the non-canonical NF-κB cascade. IAP proteins can control cell death signaling pathways via distinct mechanisms, e.g. by inhibiting caspases, by preventing the assembly of a cytosolic multiprotein complex that contains, among other proteins, RIP1 and signals to cell death and by stimulating NF-κB activation and NF-κB-dependent upregulation of cytotoxic cytokines [4]. Thus, IAP proteins are not only involved in the regulation of apoptosis, but also in the control of necroptosis, an alternative, non-apoptotic form of programmed cell death [6].

IAP proteins can contribute to radiation resistance, since they block cell death pathways at several levels and are expressed at high levels in various cancers [7]. In addition, XIAP expression levels have been reported to be upregulated in response to irradiation, resulting in resistance to radiation-induced cell death [8,9]. Against the background that IAP proteins are critical regulators of cell death and survival in cancer cells, the therapeutic targeting of IAP proteins has attracted considerable attention over the last decades. More specifically, several approaches have been developed to neutralize IAP proteins in human cancers in order to lower the threshold for the induction of cell death or to directly engage the apoptotic program. One of the most promising strategies has been the development of small-molecule inhibitors of IAP proteins that mimick the endogenous IAP protein antagonist second mitochondrial-derived activator of caspases (Smac), a mitochondrial protein that is released from the mitochondrial intermembrane space into the cytosol upon the induction of apoptosis [4]. Smac binds to and neutralizes IAP proteins including XIAP, cIAP1 and cIAP2. Smac mimetics that neutralize XIAP, ciAP1 and cIAP2, are considered to exert their maximal antitumor activity by targeting XIAP as well as cIAP proteins [10]. The Smac mimetic-mediated neutralization of XIAP results in increased caspase activation and caspase-mediated apoptosis, while the inhibition of cIAP proteins can engage an autocrine/paracrine cell death loop via Tumor Necrosis factor (TNF)α/TNF receptor (TNFR)1 signaling. This autocrine TNFα loop is engaged upon treatment with Smac mimetics via depletion of cIAP proteins by the proteasome which in turn stimulates non-canonical NF-κB signaling and subsequent upregulation of NF-κB target genes such as TNFα. Subsequent binding of TNFα to TNFR1 can engage cell death pathways in the presence of Smac mimetics that cause depletion of cIAP proteins. Besides this autocrine/paracrine TNFα loop that signals to cell death, death receptor 5 (DR5) has been identified as a critical mediator of Smac mimetic-induced apoptosis [11].

### Genetic inhibition of IAP proteins for radiosensitization

Several genetic approaches have been described to neutralize the antiapoptotic function of IAP proteins in order to enhance the sensitivity of cancer cells for radiotherapy. One possibility is ectopic expression of Smac. Overexpression of full-length or the mature form of Smac has been described to substantially enhance γ-irradiation-mediated apoptosis of neuroblastoma, glioblastoma or pancreatic carcinoma cells and also suppressed clonogenic, long-term survival [12]. Mechanistic studies showed that ectopic expression of Smac did not alter the DNA damage/repair response or activation of cellular stress programs including NF-κB or activation of DNA damage checkpoint regulators such as p53 and p21 in response to γ-irradiation [12]. Instead, overexpression of Smac potentiated the γ-irradiation-stimulated activation of caspases, loss of mitochondrial membrane potential and cytochrome c release as well as activation of caspases [12]. Similarly, RNA interference-mediated silencing of XIAP significantly increased γ-irradiation-mediated apoptosis of pancreatic carcinoma cells [13]. Also in breast carcinoma, overexpression of full-length or the mature form of Smac increased the apoptosis-induced potential of irradiation [14]. Moreover, increased radiosensitivity was reported in chondrosarcoma cells, laryngeal carcinoma, colorectal carcinoma or lung carcinoma upon genetic silencing of XIAP [15-19]. It is interesting to note that the radiation-mediated apoptosis upon XIAP knockdown was even more pronounced in mutated p53 lung carcinoma cells compared to wild-type p53 cells [18], indicating that XIAP targeting might be especially effective for radiosensitization in lung carcinoma cells with mutated p53 [18].

### Pharmacological inhibition of IAP proteins for radiosensitization

In addition to the above mentioned genetic approaches to neutralize IAP proteins in human cancers in order to eliminate a key control point in radioresistance, a range of small-molecule inhibitors of IAP proteins have been developed over the last years. For example, small-molecule inhibitors of IAP proteins were reported to significantly enhance γ-irradiation-mediated apoptosis and loss of viability of glioblastoma cells [20]. In addition, IAP inhibitors cooperated with γ-irradiation to suppress long-term clonogenic survival of glioblastoma cells [20]. This IAP inhibitor-mediated radiosensitization was shown to involve increased mitochondrial outer membrane permeabilization, caspase activation and caspase-mediated apoptotic cell death [20]. In addition to established glioblastoma cell lines, IAP inhibitors were also able to sensitize primary cultured glioblastoma cells derived from glioblastoma samples as well as glioblastoma-initiating stem-like cancer cells for cell death upon γ-irradiation [20]. In sharp contrast, IAP inhibitors failed to increase radiotoxicity in several non-malignant cells of the central nervous system [20]. These results point to some tumor selectivity of IAP inhibitor-mediated radiosensitization in glioblastoma. Furthermore, the small-molecule Smac mimetic BV6 was shown to be a potent sensitizer of glioblastoma cells for γ-irradiation-mediated apoptosis [21]. Interestingly, NF-κB turned out to exert proapoptotic functions in this model of apoptosis, as it was shown to be critically required for Smac mimetic-imposed radiosensitization of glioblastoma cells [21]. Genetic inhibition of NF-κB by overexpression of a dominant-negative superrepressor IκBα rescued BV6- and γ-irradiation-mediated apoptosis underlining that NF-κB is required for cell death induction [21]. Similarly, inhibition of NF-κB by overexpression of a kinase dead mutant of IKKβ abolished the BV6-mediated increased cell death upon γ-irradiation [21]. The potency of this combination therapy of BV6 and γ-irradiation was documented by calculation of combination index (CI) which revealed a high degree of synergy [21]. The clinical relevance was emphasized by parallel experiments using primary glioblastoma specimens [21], showing that BV6 similarly enhanced γ-irradiation-mediated cell death in primary cultured glioma cells as well as in glioblastoma-initiating cancer stem cells [21]. Furthermore, the Smac mimetic LBW242 was described to enhance the cytotoxic activity of radiotherapy of glioblastoma cells. *In vivo* studies in a glioblastoma xenograft mouse model also showed a synergistic suppression of tumor growth by co-administration of LBW242 radiation and Temozolomide (TMZ) [22]. These results demonstrate that the anti-glioma activity of radiotherapy and TMZ as state-of-the-art chemotherapy can be potentiated by the addition of Smac mimetic. Furthermore, small-molecule inhibitors of IAP proteins were shown to sensitize pancreatic carcinoma cells for γ-irradiation-triggered apoptosis [13]. In contrast, these IAP inhibitors did not affect γ-irradiation-induced apoptotic cell death of non-malignant fibroblasts, pointing to some tumor selectivity [13]. This potentiation of radiation-induced apoptosis by IAP inhibition was also documented in an independent study [23]. Here, the XIAP antagonist compounds 1396-11 and 1396-12 were shown to increase radiosensitivity of pancreatic carcinoma *in vitro* and in a subcutaneous xenograft model *in vivo* [23]. The potential of Smac mimetics to confer increased radiosensitivity was also documented in a range of carcinoma types including lung carcinoma, breast carcinoma, head and neck squamous cell carcinoma, prostate carcinoma and colorectal carcinoma [22,24-27]. In breast carcinoma, the Smac mimetic SM164-mediated radiosensitization was shown to involve both extrinsic and intrinsic apoptosis pathways including activation of caspases [24]. Mechanistic studies in head and neck squamous cell carcinoma revealed that Smac mimetic SM164-mediated radiosensitization was linked to NF-κB activation and secretion of TNFα, followed by activation of caspases and caspase-mediated apoptosis [25]. The Smac mimetic-mediated radiosensitization was also confirmed in a tumor xenograft model of head and neck squamous cell carcinoma with minimal toxicity [25]. Similarly, the Smac mimetic SH130 was shown to potentiate the growth-inhibitory effects of radiotherapy in a mouse xenograft model of prostate cancer without increasing systemic toxicity [26]. In mechanistic terms, it is interesting to note that the Smac mimetic SH130 was reported to reduce radiation-mediated activation of NF-κB in prostate carcinoma cells, pointing to a context-dependent role of NF-κB in Smac mimetic-mediated radiosensitization [26]. Besides small-molecule inhibitors of IAP proteins, antisense oligonucleotides targeting XIAP were recorded to increase radiosensitivity in preclinical models of lung cancer [28]. XIAP antisense oligonucleotides not only enhanced irradiation-induced apoptosis *in vitro* but also potentiated the growth delay upon irradiation in a xenograft mouse model *in vivo* [28].

## Conclusions

The concept to neutralize IAP proteins for radiosensitization of human cancers has proved to be a promising strategy for radiosensitization of human cancers in various preclinical models *in vitro* and *in vivo*. Since several distinct Smac mimetics are currently being evaluated in early clinical trials, both as monotherapy as well as in combination with other cytotoxic therapies including chemotherapy, it is feasible that this concept can in principle be translated into a clinical application. To this end, it will be important to identify biomarkers that might help to select those groups of patients that will benefit most from Smac mimetic-based combination therapies with radiotherapy. Taken together, targeting IAP proteins, e.g. by small-molecule inhibitors such as Smac mimetics, represents a promising avenue for future research in order to enhance the efficacy of radiotherapy.
